# Quality of Reporting and Study Design of CKD Cohort Studies Assessing Mortality in the Elderly Before and After STROBE: A Systematic Review

**DOI:** 10.1371/journal.pone.0155078

**Published:** 2016-05-11

**Authors:** Anirudh Rao, Katharina Brück, Shona Methven, Rebecca Evans, Vianda S. Stel, Kitty J. Jager, Lotty Hooft, Yoav Ben-Shlomo, Fergus Caskey

**Affiliations:** 1 UK Renal Registry, Bristol, United Kingdom; 2 Southmead Hospital, Bristol, United Kingdom; 3 School of Social and Community Medicine, University of Bristol, Bristol, United Kingdom; 4 ERA-EDTA Registry, Department of Medical Informatics, Academic Medical Center - University of Amsterdam, Amsterdam, The Netherlands; 5 School of Clinical Sciences, University of Bristol, Bristol, United Kingdom; 6 Dutch Cochrane Centre, Julius Center for Health Sciences and Primary Care, University Medical Center Utrecht, Utrecht, The Netherlands; Renal Division, Peking University First Hospital, CHINA

## Abstract

**Background:**

The STrengthening the Reporting of OBservational studies in Epidemiology (STROBE) statement was published in October 2007 to improve quality of reporting of observational studies. The aim of this review was to assess the impact of the STROBE statement on observational study reporting and study design quality in the nephrology literature.

**Study Design:**

Systematic literature review.

**Setting & Population:**

European and North American, Pre-dialysis Chronic Kidney Disease (CKD) cohort studies.

**Selection Criteria for Studies:**

Studies assessing the association between CKD and mortality in the elderly (>65 years) published from 1st January 2002 to 31st December 2013 were included, following systematic searching of MEDLINE & EMBASE.

**Predictor:**

Time period before and after the publication of the STROBE statement.

**Outcome:**

Quality of study reporting using the STROBE statement and quality of study design using the Newcastle Ottawa Scale (NOS), Scottish Intercollegiate Guidelines Network (SIGN) and Critical Appraisal Skills Programme (CASP) tools.

**Results:**

37 papers (11 Pre & 26 Post STROBE) were identified from 3621 potential articles. Only four of the 22 STROBE items and their sub-criteria (objectives reporting, choice of quantitative groups and description of and carrying out sensitivity analysis) showed improvements, with the majority of items showing little change between the period before and after publication of the STROBE statement. Pre- and post-period analysis revealed a Manuscript STROBE score increase (median score 77.8% (Inter-quartile range [IQR], 64.7–82.0) vs 83% (IQR, 78.4–84.9, p = 0.05). There was no change in quality of study design with identical median scores in the two periods for NOS (Manuscript NOS score 88.9), SIGN (Manuscript SIGN score 83.3) and CASP (Manuscript CASP score 91.7) tools.

**Limitations:**

Only 37 Studies from Europe and North America were included from one medical specialty. Assessment of study design largely reliant on good reporting.

**Conclusions:**

This study highlights continuing deficiencies in the reporting of STROBE items and their sub-criteria in cohort studies in nephrology. There was weak evidence of improvement in the overall reporting quality, with no improvement in methodological quality of CKD cohort studies between the period before and after publication of the STROBE statement.

## Introduction

Chronic kidney disease (CKD) is a complex chronic condition, and in recent years has emerged as a major public health problem[1, 2]. CKD has been termed a “Geriatric Giant”, as this disproportionately affects the elderly and is assuming epidemic proportions. Also with increasing life expectancy, patients are surviving longer with chronic conditions including CKD [3]. With the increasing burden of CKD, research of treatments developed to improve morbidity and mortality is vital [4]. Randomised controlled trials (RCTs) indisputably hold many advantages over observational studies, but owing to ethical or other considerations, may be difficult or impossible to undertake[5–7]. In nephrology there has not only been a lack of RCTs, but a large proportion of these RCTs have had negative or null findings[6]. Observational studies can provide extremely valuable additional evidence, and when rigorously undertaken may yield similar results as RCTs at far lower expense[8–11].

Standardized reporting of cohort studies is crucial for the evaluation of the merits and flaws of observational research. Inadequate reporting is associated with potentially biased estimates of treatment effects and limits the assessment of a study’s strengths, weaknesses and generalizability[12]. In order to address this, the STrengthening the Reporting of OBservational Studies in Epidemiology (STROBE) initiative developed recommendations on what should be incorporated in a precise and thorough report of an observational study. The STROBE statement and checklist were published in October 2007[13, 14]. These reporting guidelines were envisioned to make issues such as confounding, bias, and generalizability more ostensible. In the long term, this would improve the methodology of studies by increased awareness of these issues for researchers designing a new study[15, 16].

The scientific value and reliability of the conclusions drawn from a study are determined to a major extent by the quality of the study design[17]. A variety of tools currently exist to assess the risk of bias (methodological quality) of observational studies, and are employed when undertaking a systematic review. These include quality scales, simple checklists, or checklists with a summary judgment for assessment of the risk of bias[18].

The objectives of this review were (a) to determine whether the publication of the STROBE statement is associated with an improvement in the reporting quality of cohort studies assessing mortality in elderly patients with CKD; and (b) to determine whether the publication of the STROBE statement is associated with a decrease in risk of bias (improvement in the methodological quality) of cohort studies assessing mortality in elderly patients with CKD.

## Materials and Methods

### Data selection

A systematic literature search was performed in Medline and Embase using the OvidSP interface to identify all papers describing pre-dialysis CKD cohort studies in the elderly (> 65 years) where mortality was reported as an outcome. This systematic review was conducted a part of the background preparation for the EQUAL study which is an international (European) multicentre prospective observational cohort study looking at the timing of the start of dialysis in elderly patients (≥65 years) with estimated glomerular filtration rate (eGFR) of ≤ 20mls/min and therefore the review is restricted to CKD cohort studies in the elderly[19]. The search query is presented in Item A in S1 File (available as online supplementary material).

Papers published between 1^st^ January 2002 and 31^st^ December 2013 were included, as the KDOQI Clinical Practice Guidelines for Chronic Kidney Disease: Evaluation, Classification, and Stratification were published in 2002[20]. Only articles published in English were considered for the purposes of the review. The initial search strategy yielded more than 10,000 hits, hence the number of studies were reduced by restricting the search to European and North American studies. Each article was double sifted at title, abstract and full text stage using predefined study inclusion and exclusion criteria. Any disagreements about inclusion were resolved by discussion.

The systematic review aimed to cover reporting and design of observational studies before and after the publication of the STROBE statement which was published in October 2007. We assessed reporting and methodological quality during two time periods: before STROBE between 1/1/2002-31/12/2007 and after STROBE 1/10/2008-31/12/2013, allowing a one-year run-in period. By excluding publications in the immediate twelve months post-STROBE we allowed a period of one year for submission, revision and publication of research adhering to the new guidelines.

### Data extraction

The reporting of the selected studies was assessed using the STROBE checklist itself, and the methodological quality assessed using three tools. Thirteen of the 22 STROBE checklist items were assessed with 2 to 6 questions per item generating 55 questions. The STROBE checklist is presented in Table A in S1 File (available as online supplementary material). These could be answered as “yes,” “partly,” “no,” “unclear,” or “not applicable”. We used similar methodology to that reported in the publication by Langan et al[21].

To assess methodological quality, the articles were scored on the Newcastle Ottawa Scale (NOS). At the time this study was designed NOS was recommended by Cochrane for evaluating the risk of bias in observational studies for inclusion in systematic reviews [22, 23]. The articles were also scored using the Scottish Intercollegiate Guidelines Network (SIGN) checklist for cohort studies[24], and Critical Appraisal Skills Programme (CASP) cohort studies checklist[25] to estimate concurrent validity of NOS tool. These three checklists were chosen because they were simple checklists without an additional summary judgement[26].

The eligible papers that were identified by the sifting process were each scored using the STROBE, NOS, SIGN and CASP checklists by two reviewers. Where there was disagreement between reviewers, consensus was reached by discussion.

### Outcome measure

Quality of study reporting was calculated by specific STROBE items and at a manuscript level. A STROBE question score (SQS) was calculated; the number of publications in a period that adequately reported a question divided by the number of publications in which this question was applicable, expressed as a percentage (item analysis). A Manuscript STROBE score (MSS) was calculated for every manuscript; the number of questions (maximum of 55 questions) adequately reported in the publication divided by the number of applicable questions, expressed as a percentage (manuscript analysis).

Similarly, to assess the quality of study design the manuscript NOS score (MNOS), manuscript SIGN score (MSiS) and manuscript CASP score (MCAS) were calculated; the number of questions adequately addressed (in each appraisal tool) divided by the number of applicable items, expressed as a percentage in order to facilitate comparison.

### Data Analysis

Comparison between pre and post-period SQS was performed by calculating the risk (proportion) difference between the two groups using the Wald test and respective 95% confidence intervals, with Benjamini and Hochberg adjusted p values (False Discovery Rate) to control for multiple testing[27]. MSS, MNOS, MSiS and MCAS were reported as a median with respective interquartile range (IQR). Pre- and post-period median MSS, MNOS, MSiS and MCAS were compared using the Mann-Whitney (MW) test. Despite excluding articles published for a period of 1 year after introduction of STROBE, this could potentially have been insufficient for uptake and penetration of new information. Therefore a spline linear regression model was used to determine the impact of STROBE over time[28]. Sub-group analyses of MSS were carried out restricting articles to those published in nephrology journals, STROBE endorsing and non-endorsing journals and by journal impact factor in the year that the article was published. Sensitivity analyses were carried out by excluding the outlying MSS if any data points were less than 1.5 interquartile ranges (IQRs) below the first quartile or above the third quartile (< Q1–1.5×IQR or > Q3 + 1.5×IQR). Simple and weighted kappa statistics were used to compare agreement between reviewers for the NOS, SIGN and CASP checklists. All tests were two-tailed, and p values, < 0.05 were considered statistically significant. Data were analysed using STATA v13.1 (College Station, TX, USA) and SAS v9.3 (SAS Institute, Cary, NC, USA) software.

### Reporting

The study has been reported in accordance with PRISMA reporting guidelines.

## Results

### Reporting Quality

**Fig 1:**
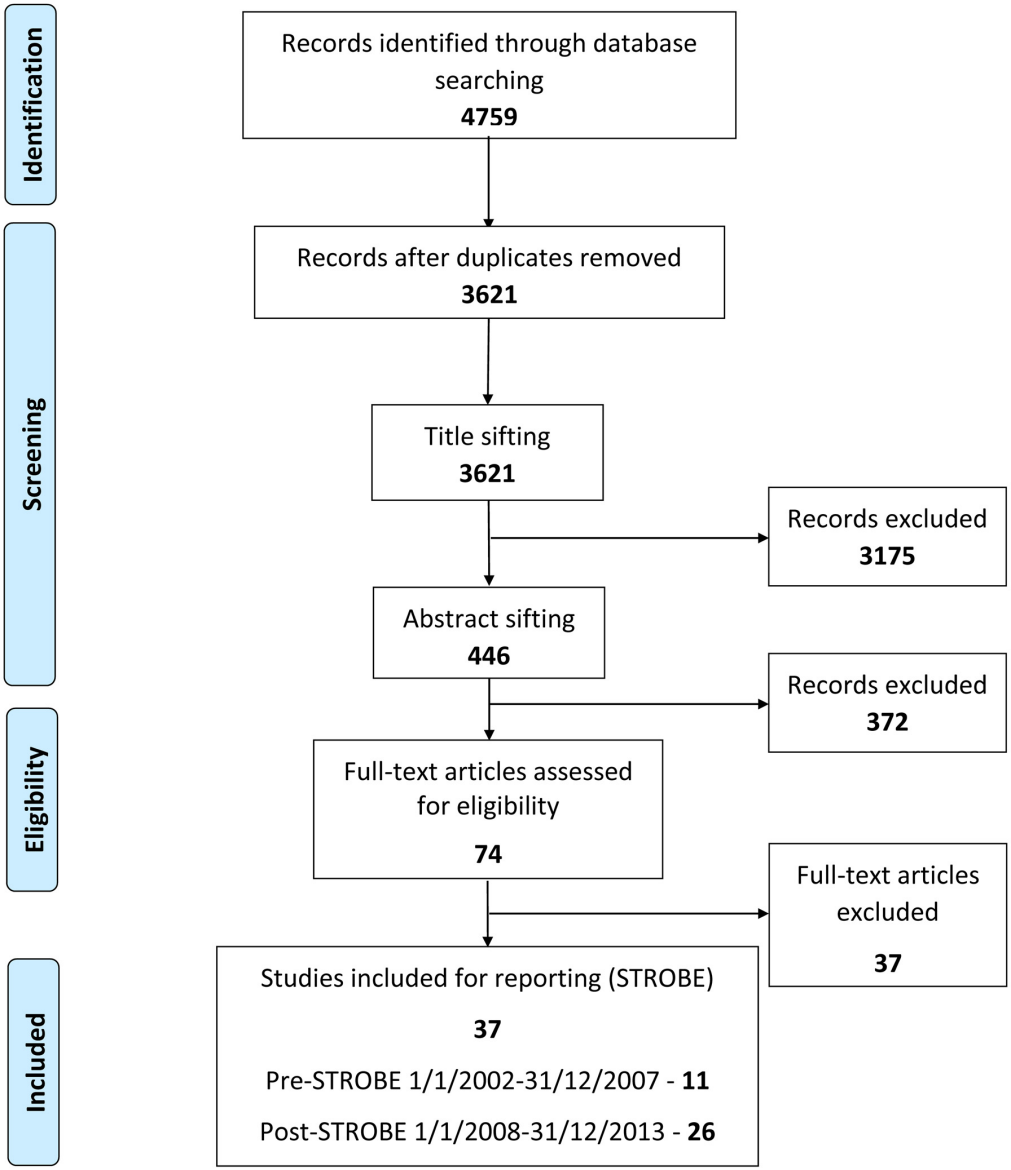
PRISMA flow diagram.

Fig 1 shows the flow diagram of exclusions. Of the 3621 articles initially identified by the Medline and Embase search, 3584 (98.9%) were excluded after the sifting process (Fig 1). Only 37 articles met the pre-defined selection criteria for the scoring stage of the review during the inclusion period. Of these 37 articles, 11 were in the pre-STROBE era (1/1/2002-31/12/2007) & 26 in the post-STROBE period (1/10/2008-31/12/2013). Twenty-two of these articles were published in nephrology and 15 in other medical journals. The list of articles considered at the scoring stage of the study is provided in Item B in S1 File (available as online supplementary material).

Table 1 summarises the STROBE, NOS, SIGN and CASP scores for each of the articles in the pre and post-STROBE period. In most cases, reporting quality (STROBE) and methodological quality (NOS, SIGN and CASP) correlated well. However, in some articles methodological quality scored highly with a low score for reporting and vice versa.

**Table 1:** Summary of pre and post-STROBE period Manuscript STROBE score (MSS), Manuscript NOS score (MNOS), Manuscript SIGN score (MSiS) & Manuscript CASP score (MCAS) by article. The citations for the manuscripts are listed in online supplementary material (Item B in S1 File).

Publication date	Journal	Study Reporting	Study Design		
		MSS	MNOS	MSiS	MCAS
**Pre-STROBE**					
Dec-02	Journal of American College of Cardiology	76.5	55.6	22.2	33.3
Jun-03	American Journal of Kidney Diseases	77.8	88.9	91.7	100.0
Oct-04	Journal of the American Society of Nephrology	66.7	66.7	50.0	33.3
Apr-05	The Journal of the American Medical Association	88.7	88.9	100.0	100.0
Sep-05	Journal of the American Society of Nephrology	64.7	88.9	80.0	83.3
Dec-05	Journal of the American Society of Nephrology	84.6	66.7	61.5	83.3
Nov-06	British Medical Journal	82.0	100.0	100.0	91.7
Jul-07	Renal Failure	49.1	100.0	88.9	100.0
Jul-07	Journal of the American Society of Nephrology	80.4	100.0	100.0	100.0
Nov-07	Nephrology Dialysis Transplantation	51.1	100.0	80.0	91.7
Dec-07	Archives of Internal Medicine	77.8	88.9	83.3	91.7
**Post-STROBE**					
Nov-08	Nephrology Dialysis Transplantation	83.0	88.9	83.3	100.0
Dec-08	Nephrology Dialysis Transplantation	84.9	77.8	90.0	83.3
Feb-09	Journal of the American Society of Nephrology	72.9	100.0	83.3	91.7
Apr-09	American Journal of Kidney Diseases	90.0	100.0	69.2	100.0
Jul-09	American Journal of Kidney Diseases	83.7	100.0	100.0	91.7
Jul-09	Clinical Journal of the American Society of Nephrology	75.0	37.5	25.0	33.3
Dec-09	Journal of American Geriatric Society	78.4	100.0	87.5	100.0
Jul-10	Nephrology Dialysis Transplantation	77.6	100.0	90.9	91.7
Oct-10	Journal of Nephrology	39.6	100.0	100.0	100.0
Sep-10	Journal of General Internal Medicine	83.0	77.8	54.5	41.7
Nov-10	Rejuvenation Research	73.6	88.9	80.0	91.7
Sep-11	Clinical Journal of the American Society of Nephrology	92.2	66.7	90.9	75.0
Jan-12	Nefrologia	50.0	77.8	90.9	91.7
Feb-12	Age and Ageing	83.0	100.0	91.7	100.0
Apr-12	Nephrology Dialysis Transplantation	87.8	88.9	91.7	100.0
Apr-12	The American Journal of Medicine	79.6	100.0	83.3	83.3
May-12	Journal of American Geriatric Society	83.0	88.9	91.7	100.0
May-12	Nephrology Dialysis Transplantation	88.0	100.0	66.7	75.0
Jun-12	The Journal of the American Medical Association	84.9	88.9	66.7	66.7
Jun-12	Clinical Journal of the American Society of Nephrology	83.0	88.9	76.9	83.3
Jul-12	Journal of American Geriatric Society	79.6	88.9	100.0	91.7
Dec-12	Family Practice	84.6	77.8	63.6	83.3
Feb-13	American Journal of medicine	83.0	88.9	75.0	100.0
Apr-13	BMC Nephrology	84.6	100.0	70.0	100.0
May-13	BMC Nephrology	83.0	100.0	88.9	83.3
Sep-13	Clinical Journal of the American Society of Nephrology	90.2	100.0	80.0	66.7

Some of the STROBE question scores showed a ceiling effect as they were already at a maximum level in the pre-STROBE period and could therefore only remain static or decline. Others saw improvements over the period such as “choice of quantitative groups” (30% vs 71%, p = 0.02), “addressing of losses to follow up” (0% vs 36%, p < 0.001), “description of and carrying out sensitivity analysis” (18% vs 58%, p = 0.01 & 18% vs 65%, p = 0.002) and “usage of flow diagram” (0% vs 19%, p = 0.01). However, after adjusting for multiple testing, the change in only two items’ scores remained unlikely to be due to chance; “addressing of losses to follow up” (p = 0.02) and “carrying out sensitivity analysis” (p = 0.04). The majority of STROBE questions showed little improvement between the two periods. Some critical questions, such as hypothesis specification and those important to interpretation of study validity such as sample size estimation, addressing missing data, addressing loss to follow up, reason for non-participation and usage of flow diagram continue to be under reported with less than 50% reporting these items in both periods. Details regarding the reporting of the 55 STROBE items in the 37 included cohort studies are shown in Table 2.

**Table 2:** Median STROBE QUESTION SCORE (SQS), Difference (95% CI) with p value of the 55 data items (22 items were further sub-divided to 55 questions in total) in 37 CKD cohort studies, by publication period.

Item number	Data Items	Pre-STROBE	Post-STROBE	Difference	LCI	UCI	p value	FDR	FDR*
		SQS	SQS						
**Title and Abstract**									
1A	Is the design described adequately in the title or abstract?	0.73	0.69	-0.04	-0.35	0.28	0.83	0.91	0.93
1B	Does the abstract provide an informative summary of what was done and found?	1.00	1.00	0.00	-	-	-	-	-
**Introduction**									
2	Is the scientific background and rationale for the investigation reported?	1.00	1.00	0.00	-	-	-	-	-
3A	Are any pre specified hypotheses reported?	0.18	0.23	0.05	-0.23	0.33	0.73	0.91	0.93
3B	Are the objectives reported?	0.73	0.96	0.23	-0.04	0.51	0.09	0.62	0.64
**Methods**									
4	Are the key elements (ie, retrospective/prospective, cohort/cross-sectional) of the study design presented early in the paper?	1.00	0.92	-0.08	-0.18	0.03	0.14	0.62	.
5A	Are the settings reported?	0.91	1.00	0.09	-0.08	0.26	0.29	0.62	0.74
5B	Are the locations reported?	1.00	0.88	-0.12	-0.24	0.01	0.07	0.52	.
5C	Are relevant dates including periods of recruitment reported?	1.00	0.96	-0.04	-0.11	0.04	0.31	0.62	.
5D	Are relevant dates including periods of exposure reported?	1.00	0.93	-0.07	-0.21	0.06	0.30	0.62	.
5E	Are relevant dates including periods of follow-up reported?	0.91	0.96	0.05	-0.13	0.24	0.58	0.81	0.87
5F	Are relevant dates including periods of data collection reported?	0.73	0.92	0.20	-0.09	0.48	0.17	0.62	0.74
6A	Are the eligibility criteria for participants described?	1.00	0.92	-0.08	-0.18	0.03	0.14	0.62	.
6B	Are the sources of participants described?	1.00	0.92	-0.08	-0.18	0.03	0.14	0.62	.
6C	Are the methods of selection described?	1.00	0.96	-0.04	-0.11	0.04	0.31	0.62	.
6D	Are the methods of follow-up described?	0.91	0.96	0.05	-0.13	0.24	0.579	0.81	0.87
6E	If it is a matched study, are the matching criteria and the numbers of exposed and unexposed described?	0.00	1.00	1.00	-	-	-	-	-
7A	Are all outcomes described if applicable?	0.91	0.96	0.05	-0.13	0.24	0.58	0.81	0.87
7B	Are all exposures described if applicable?	1.00	1.00	0.00	-	-	-	-	-
7C	Are all predictors described if applicable?	0.91	1.00	0.09	-0.08	0.26	0.29	0.62	0.74
7D	Are potential confounders described?	0.91	0.92	0.01	-0.18	0.21	0.89	0.93	0.94
7E	Are all effect modifiers described?	0.73	0.80	0.07	-0.23	0.38	0.64	0.86	0.91
7F	Are diagnostic criteria described if applicable?	0.80	0.92	0.12	-0.15	0.39	0.40	0.73	0.84
8A	Are the sources of data and details of methods of measurement given for each variable of interest?	0.91	0.85	-0.06	-0.28	0.16	0.57	0.81	0.87
8B	If there is more than 1 group, are the measurement methods comparable?	0.75	1.00	0.25	-0.17	0.67	0.25	0.62	0.74
9	Was there any effort to address potential sources of bias?	0.91	0.92	0.01	-0.18	0.21	0.89	0.93	0.94
10	Did they describe how the study size was determined?	0.00	0.04	0.04	-0.04	0.13	0.31	0.62	0.74
11A	Did they describe how quantitative variables were handled in the analysis?	0.80	0.80	0.00	-0.29	0.29	1	1	1
11B	Did they describe which groupings were chosen for quantitative variables?	0.90	0.83	-0.07	-0.31	0.17	0.58	0.81	0.87
11C	Did they describe why quantitative groups were chosen?	0.30	0.71	0.41	0.07	0.75	**0.02**	0.17	0.14
12A	Did they describe all statistical methods including those to deal with confounding?	0.91	1.00	0.09	-0.08	0.26	0.29	0.62	0.74
12B	Did they describe methods to examine subgroups and interactions?	0.73	0.72	-0.01	-0.32	0.31	0.96	0.98	0.99
12C	Did they explain how missing data were addressed?	0.27	0.38	0.11	-0.21	0.43	0.50	0.81	0.87
12D	Did they explain if applicable how losses to follow-up were addressed?	0.00	0.36	0.36	0.16	0.56	**<0.001**	**0.02**	**0.02**
12E	Did they describe any sensitivity analysis?	0.18	0.58	0.40	0.10	0.69	**0.01**	0.14	0.12
**Results**									
13A	Did they report the numbers of individuals at each stage of the study numbers potentially eligible, examined for eligibility, confirmed eligible, included in the study, and completed follow-up and were analysed?	0.64	0.81	0.17	-0.15	0.49	0.30	0.62	0.74
13B	Did they give reasons for nonparticipation at each stage?	0.30	0.48	0.18	-0.18	0.53	0.33	0.64	0.75
13C	Did they use a flow diagram if appropriate?	0.00	0.19	0.19	0.04	0.34	**0.01**	0.15	0.13
14A	Did they give the characteristics of study participants (eg, demographic, clinical, and social) and information on exposures and potential confounders?	1.00	1.00	0.00	-	-	-	-	-
14B	Did they indicate the number of participants with missing data for each variable of interest?	0.27	0.15	-0.12	-0.42	0.18	0.43	0.74	0.85
14C	Did they summarize follow-up time (average and total amount)?	0.91	1.00	0.09	-0.08	0.26	0.29	0.62	0.7394
15A	Did they report numbers of outcome measures over time?	1.00	1.00	0.00	-	-	-	-	-
15B	Did they report summary measures over time?	0.82	0.92	0.10	-0.15	0.35	0.41	0.73	0.84
16A	Did they give unadjusted estimates and, if applicable, confounder-adjusted estimates and their precision (eg, 95% confidence interval)?	0.82	0.92	0.03	-0.24	0.29	0.84	0.91	0.93
16B	Did they detail which confounders were adjusted for and why they were included?	0.90	0.92	0.02	-0.19	0.24	0.83	0.91	0.93
16C	Did they report category boundaries when continuous variables were categorized?	0.90	0.88	-0.03	-0.25	0.20	0.83	0.91	0.93
16D	Did they, if relevant, consider translating estimates of relative risk into absolute risk for a meaningful period?	0.33	0.26	-0.07	-0.43	0.28	0.69	0.90	0.93
17A	Did they report on other analyses done, eg, analysis of subgroups or interactions?	0.64	0.85	0.21	-0.11	0.53	0.19	0.62	0.74
17B	Did they do a sensitivity analysis?	0.18	0.65	0.47	0.18	0.76	**0.002**	**0.04**	**0.03**
**Discussion**									
18	Did they summarize key results with reference to study objectives?	0.82	1.00	0.18	-0.05	0.41	0.12	0.62	0.70
19	Did they discuss the limitations of the study taking into account potential sources of bias or imprecision (including discussion of the magnitude of any potential sources of bias)?	0.91	1.00	0.09	-0.08	0.26	0.29	0.62	0.74
20	Did they give a cautious overall interpretation of results considering objectives, limitations, multiplicity of analyses, results from similar studies, and other relevant evidence?	1.00	1.00	0.00	-	-	-	-	-
21	Did they discuss the generalizability (external validity) of the study results?	0.73	0.76	0.04	-0.27	0.35	0.80	0.91	0.93
**Other Information**									
22A	Did they give the source of the funding in the present study and, if applicable, for the original study on which the present article is based?	0.73	0.77	0.03	-0.28	0.34	0.84	0.91	0.93
22B	Did they give the role of the funders in the present study and, if applicable, for the original study on which the present article is based?	0.36	0.46	0.09	-0.25	0.44	0.60	0.81	0.87

* False Discovery Rate (FDR) calculated excluding questions which had 100% completeness in the pre-STROBE phase.

Pre- and post-period analyses revealed an increase in MSS (median score 77.8 (IQR, 64.7–82.0) vs 83 (IQR, 78.4–84.9), p = 0.04) (see Table 3). Any pre-STROBE period articles with MSS scores less than 47.4 and post-STROBE period less than 69 were considered to be outliers. Excluding outliers, the improvement in the MSS between the two periods showed a stronger statistical relationship (p = 0.01). The results were essentially unchanged when restricted to nephrology journals or stratified by STROBE endorsing or non-endorsing journals, though there was less statistical power to test for differences. Journals with impact factor < 5 saw greater change over the two periods when compared to journals with impact factor ≥ 5 but given the overlap in the confidence intervals this may have occurred by chance.

**Table 3:** Summary of quality of reporting as assessed using the Manuscript STROBE Score (MSS).

	Pre-STROBE		Post-STROBE		
	N	median MSS (IQR)	N	median MSS (IQR)	p value
All Journals	11	77.8 (64.7–82.0)	26	83 (78.4–84.9)	0.04
All Journals (excluding outliers) *	11	77.8 (64.7–82.0)	24	83 (79.6–84.9)	0.01
Nephrology Journals	6	72.3 (64.7–80.4)	16	83.4 (76.3–87.9)	0.09
STROBE endorsing Journals (3)	2	79.9 (77.8–82)	3	90 (83.7–90.9)	0.08
Non-STROBE endorsing Journals (13)	9	76.5 (64.7–80.4)	23	83 (77.6–84.6)	0.10
Impact FACTOR < 5	3	51.1 (49.1–77.8)	16	83 (78–84.6)	0.06
Impact FACTOR ≥ 5	8	79.1 (71.6–83.3)	10	83.4 (79.6–90.0)	0.13

* Excluding articles that were less than 1.5 interquartile ranges (IQRs) below the first quartile (< Q1–1.5×IQR). Pre-STROBE = 47.4 & Post-STROBE = 69

**Fig 2:**
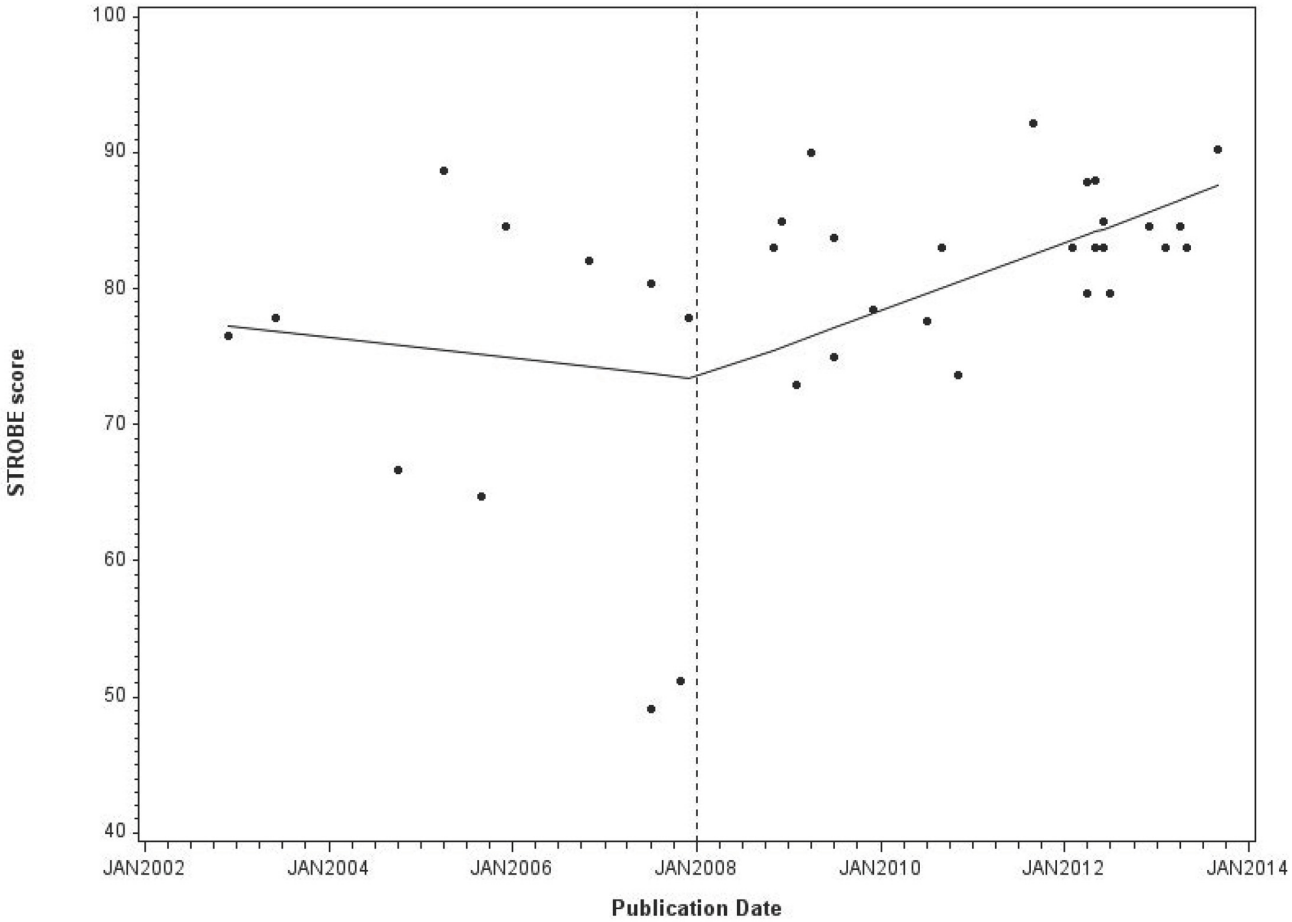
Time series of Manuscript STROBE scores (MSS) from spline linear regression models.

Time series analysis of MSS showed that there was a significant improvement in the quality of reporting in the latter three years (1/1/11 to 31/12/13) when compared to the first three years (1/10/2008 to 31/12/2010) after the introduction of the STROBE statement (Table 4). Longitudinal analysis of the MSS using a spline linear regression model (Fig 2), having excluded outliers, suggested a turning point in 2008 with a slight negative trend in the pre-STROBE period (coefficient—0.06, SE 0.11) and a positive slope in the post-STROBE period (coefficient 0.21 SE 0.05) but this may have occurred by chance (Slope change coefficient 0.27, SE 0.16; p value = 0.10).

**Table 4:** Quality of the reporting of observational studies as assessed using the Manuscript STROBE score (MSS) over time.

	Pre-STROBE publication	Immediate Post-STROBE publication	Late Post-STROBE publication	p value	p value
	(period 1)	(period 2)	(period 3)	period	period
	1/1/2002 to 31/12/2007	1/10/2008 to 31/12/2010	1/1/11 to 31/12/13	1 vs 2	1 vs 3
**N**	11	10	14		
**Median MSC**	77.8	80.7	83.8	0.23	0.003
**IQR**	64.7–82.0	75–83.7	83–87.8		

### Methodological quality (study design); comparison in the pre- and post-STROBE period

**Fig 3:**
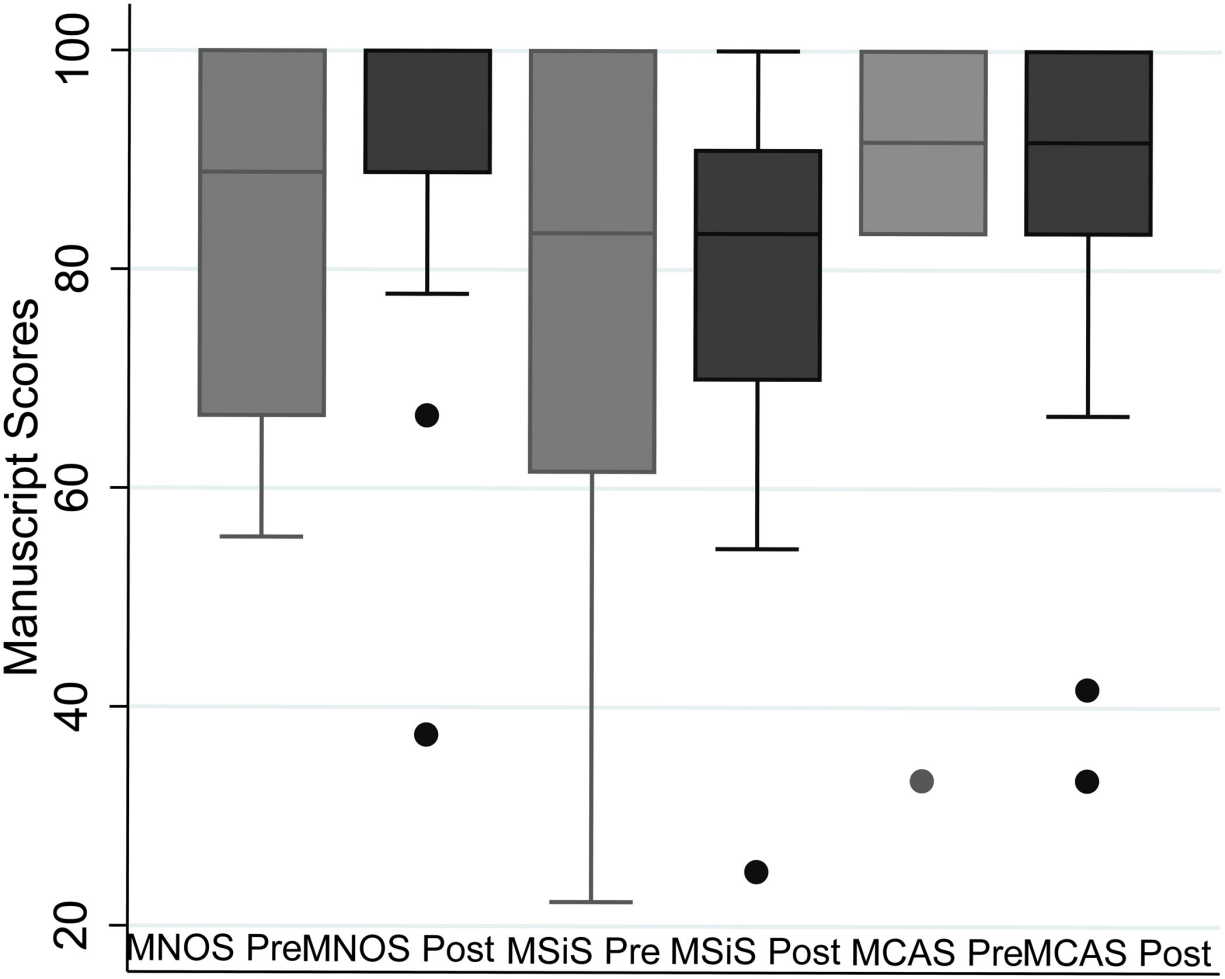
Box plot summarising methodological quality of the studies in the Pre and Post-STROBE period as assessed using the NOS, CASP and SIGN.

We found no evidence for any change in the methodological quality of studies in the pre and post-STROBE period using the Newcastle Ottawa Scale (NOS) (median MNOS 88.9% [IQR, 66.7–100] vs 88.9% [IQR, 88.9–100], p = 0.51), Scottish Intercollegiate Guidelines Network (SIGN) (median MSiS 83.3% [IQR, 61.5–100] vs 83.3% [IQR, 70–90.9], p = 0.93) and Critical Appraisal Skills Programme (CASP) (median MCAS 91.7% [IQR, 83.3–100] vs 91.7% [IQR, 83.3–100], p = 0.93) (Fig 3).

### Inter-rater agreement

Agreement between raters for the NOS, SIGN and CASP tools was calculated using the simple or weighted Kappa coefficient. These were assessed at three levels: raters’ agreement on applicability, clarity (can’t say) and yes/no. The inter-rater agreement for each of the tools was overall inadequate, with the NOS tool having poor agreement between the three pairs of raters’. The CASP tool fared slightly better compared to the SIGN tool in raters’ assessment of clarity. A summary table of Kappa coefficients is included in Table B in S1 File (available as online supplementary material).

## Discussion

This systematic review assessed the impact of the publication of the STROBE statement on quality of study design and reporting of methodology. It showed that, after publication of STROBE, a large proportion of the STROBE items and sub-criteria continue to be underreported in CKD cohort studies of mortality in elderly patients. Reporting rates were lowest for hypothesis specification, usage of flow diagrams and addressing missing data. There was evidence of improvement in the reporting quality of CKD cohort studies particularly in the latter three years of the post-STROBE period, which was also seen when looking at the temporal patterns but this may have occurred by chance. We found no evidence that the quality of study design as assessed by 3 different tools NOS, SIGN and CASP had improved. However, these quality assessment tools have poor to moderate inter-rater reliability and might not be suitable for use without consensus agreement between raters.

The publication of CKD guidelines in 2002 has potentially had an impact on the volume of CKD research with approximately 2.5 times the number of studies in the post-STROBE period compared to the pre-STROBE period[20].

Inadequate reporting not only hinders critical assessment by others of the strengths and weaknesses in study design, conduct, and analysis, it affects judgement of whether and how results can be included in systematic reviews and also impacts on the reader assessment of the studies generalizability [29]. Our results are consistent with other studies assessing deficiencies in reporting of individual STROBE items such sample size, use of flow diagram and reporting of missing data [21, 30–35].

A number of studies, including a Cochrane review, have demonstrated improvements in reporting quality of randomised control trials (RCTs) after the introduction of the Consolidated Standards of Reporting Trials (CONSORT) statement with a significant improvement in journals endorsing this guideline statement[36–40]. An RCT has also shown that using reporting guidelines in the peer review process improves the quality of manuscripts[41]. Our study showed weak evidence of improvement in the quality of reporting of CKD cohort studies over time following the introduction of the STROBE statement. The improvements unfortunately fell short of the intended expectations when compared to the impact the CONSORT statement had achieved upon the reporting quality of RCTs. These results were similar to the only other study looking at quality of reporting, published in the dermatology literature. Those authors attributed the lack of improvement to the short follow up period after STROBE introduction (2008–10)[30]. However, in our study the small improvement could be attributable to the fact that the reporting of nephrology literature in the pre-STROBE period was already of a higher standard (median MSS 77.8 IQR 64.7–82.0) in comparison to dermatology literature (median score 58 IQR 46–73).

Journal endorsement of reporting guidelines has been shown to improve reporting quality of manuscripts submitted to journals[41]. Given that only two medical journals (British Medical Journal & Ageing) and one renal journal (American Journal of Kidney Diseases) included in this review had endorsed the STROBE statement, any evidence of improvement in reporting quality of cohort studies in nephrology literature is probably attributable to the penetration of STROBE statement over time rather than to its endorsement by journals[42]. The lack of improvement of reporting standards seen in the STROBE endorsing journals is not an indictment of these journals but maybe attributable to the small sample size to accurately test for differences between the groups. An important observation that was made during the process of this review was that despite studies having similar reporting standards, reflected by their similar MSS, some studies had failed to adequately report essential criteria.

For most of the articles included in this study reporting and methodological quality were well correlated, however the assessment of the methodological quality of a study is largely dependent on adequate reporting of the research. Therefore, drawing any inferences about a study’s design quality is made harder if the reporting quality is inadequate.

One of the main goals of reporting guidelines was to improve reporting clarity and not necessarily improve the quality of research, but in due course achieve it as an indirect effect. Due to interchangeable usage of the terminology ‘reporting quality’ and ‘methodological quality’, the STROBE statement has often been used inappropriately for the assessment of methodological quality of observational research[16]. There are a number of assessment tools that have been developed to assess quality and susceptibility to bias in observational studies with only half of the identified tools have described their development or validity and reliability [26]. The review by Sanderson et al highlighted the lack of a single obvious tool for assessing quality of observational epidemiological studies[26]. The bias assessment tools used in this study (NOS, SIGN and CASP) were subjective, differed by content, format and validity. The bias assessment tools identified deficiencies in the articles relating to consideration of participant’s lost to follow up (attrition bias), exposure level or prognostic factor measured only once (detection bias), and inadequate methods of outcome assessment (detection bias). However, given that the assessment of methodological quality is largely reliant on the reporting of study design, one might therefore fail to detect differences in design quality if reporting is inadequate. Also given the latency period of designing a new study, undertaking it and then publishing it, might have been simply too soon for the STROBE statement to have influenced the methodological quality of studies. The NOS tool was previously recommended by Cochrane for evaluating the risk of bias but published literature has demonstrated poor inter-rater reliability between individual reviewers[43, 44]. The results of our study are consistent with these findings as all of the three tools (NOS, SIGN and CASP) showed poor agreement between individual reviewers. The usability of a tool depends on its clarity. Moreover, the tools contain items whose scoring is subjective and dependent on reviewers’ perceptions and domain knowledge. Cochrane now recommends the ACROBAT-NRSI bias assessment tool for non-randomized studies which has been developed by members of the Cochrane Bias Methods Group and the Cochrane Non-Randomised Studies Methods Group[45, 46]. However, at the time of drafting this manuscript, this tool remains yet to be tested for consistency between individual reviewers.

A strategy to improve inter-rater agreement would be tailoring and training of reviewers prior to implementation of the tools. Due to the poor reliability of the tools demonstrated here, it should be strongly considered that each study should be assessed by at least two reviewers prior to inclusion in a systematic review/meta-analysis.

One of the strengths of our study is that we studied the impact of STROBE upon both quality of reporting and study design. The study has good internal validity as the selection and evaluation processes were independently performed by two reviewers. However, as the articles were included from one field of medicine (CKD) we must be cautious in generalising our findings to other areas. The other limitation of the study was that it only covered articles from Europe and North America. There was also an imbalance in the number of studies assessed in the two periods probably due to the KDIGO CKD guidelines which were published in 2002. This imbalance could have potentially introduced a lack of power to detect difference in quality. It was also impossible to blind the reviewers to the publication date during the sifting stage of the review, and the journal name during the review of quality which could have biased the reviewers’ assessment of quality of the study. Finally, whilst we examined a five-year period post-STROBE, it is possible that we failed to find any benefit for methodological quality due to the long latency period between designing a new study, obtaining funding, undertaking data collection, analysis and publication.

## Conclusion

This study highlights continuing deficiencies in the reporting of observational studies in the nephrology literature. However, the publication of the STROBE statement may have positively influenced the quality of some aspects of observational study reporting. There was no evidence, however, that methodological quality improved over this time period. With continued efforts from researchers and with particular focus on the domains identified as deficient by the STROBE statement and bias reporting tools, this presents an opportunity to improve the validity of observational research in nephrology. With increased awareness by authors and editors regarding compliance of manuscripts to the STROBE statement and journal endorsement of the STROBE statement, we hope that not only reporting but also the design of future studies will be improved.

## Supporting Information

S1 File **Item A:** Search strategy for systematic review. **Table A:** STROBE scoring sheet. **Item B:** List of articles included for review by date of publication. **Table B:** Summary of simple and weighted Kappa coefficient, measuring agreement between reviewers for the NOS, SIGN and CASP tool.
(DOCX)
