# Supplementary material for: Quality of Reporting and Study Design of CKD Cohort Studies Assessing Mortality in the Elderly Before and After STROBE: A Systematic Review
Source: PLoS One. 2016 May 11;11(5):e0155078. doi: 10.1371/journal.pone.0155078 (PMC4863970; doi:10.1371/journal.pone.0155078)
Supplement: S1 File — Item A: Search strategy for systematic review. Table A: STROBE scoring sheet. Item B: List of articles included for review by date of publication. Table B: Summary of simple and weighted Kappa coefficient, measuring agreement between reviewers for the NOS, SIGN and CASP tool. (DOCX) [file pone.0155078.s001.docx]

**Online supplementary material (S1 File)**

**Item A: Search strategy for systematic review**

Chronic Kidney Disease (CKD):

exp renal insufficiency, chronic/

exp Cardiovascular Diseases/ep [Epidemiology]

kidney disease*.ti,ab.

renal disease*.ti,ab.

kidney insufficienc*.ti,ab.

renal insufficienc*.ti,ab.

kidney failure .ti,ab.

kidney dysfunction*.ti,ab.

renal dysfunction*.ti,ab.

kidney impairment*.ti,ab.

renal impairment*.ti,ab.

impaired kidney function*.ti,ab.

impaired renal function*.ti,ab.

decreased kidney function*.ti,ab.

decreased renal function*.ti,ab.

chronic kidney.ti,ab.

chronic renal.ti,ab.

CKD.ti,ab.

CRD.ti,ab.

ESRD.ti,ab.

ESKD.ti,ab.

CKF.ti,ab.

Cohort:

exp cohort studies/

cohort*.pt,ti,ab.

Longitudinal*.pt,ti,ab.

Follow-up*.pt,ti,ab.

Follow up*.pt,ti,ab.

Prospective* .pt,ti,ab.

Retrospective* .pt,ti,ab.

Observational* .pt,ti,ab.

Elderly:

exp Aged/

elder* .ti,ab.

Older .ti,ab.

Oldest .ti,ab.

old age .ti,ab.

old people .ti,ab.

Geriatric .ti,ab.

Aging .ti,ab.

Ageing .ti,ab.

Frail .ti,ab.

community dwelling .ti,ab.

nursing home .ti,ab.

home for the aged .ti,ab.

homes for the aged .ti,ab.

Residents .ti,ab.

Mortality:

mortality/ or *"cause of death"/ or *fatal outcome/ or *hospital mortality/ or *mortality, premature/ or *survival rate/

exp Renal Insufficiency, Chronic/mo [Mortality]

Europe:

1. exp Europe/

2. UK.mp. [mp=title, abstract, original title, name of substance word, subject heading word, keyword heading word, protocol supplementary concept, rare disease supplementary concept, unique identifier]

3. United Kingdom.mp. [mp=title, abstract, original title, name of substance word, subject heading word, keyword heading word, protocol supplementary concept, rare disease supplementary concept, unique identifier]

4. England.mp. [mp=title, abstract, original title, name of substance word, subject heading word, keyword heading word, protocol supplementary concept, rare disease supplementary concept, unique identifier]

5. English.mp.

6. (Austria or Austrian).mp.

7. Belgium*.mp.

8. Albania*.mp.

9. Estonia*.mp.

10. Latvia*.mp.

11. Lithuania*.mp.

12. Bosnia*-Herzegovina*.mp.

13. Bulgaria*.mp.

14. Croat*.mp.

15. Hungarian*.mp.

16. Hungary.mp.

17. Macedonia*.mp.

18. (Moldova or Moldavian* or Moldovan).mp.

19. Montenegr*.mp.

20. (Poland or Polish or Pole*).mp.

21. Belarus*.mp.

22. Romania*.mp.

23. Russia*.mp.

24. Serbia*.mp.

25. Slovak*.mp.

26. Sloven*.mp.

27. Ukrain*.mp.

28. Yugoslavia*.mp.

29. (Finland or Finns or Finn or Finnish).mp.

30. (France or French).mp.

31. German*.mp.

32. (Great Britain or Great British).mp.

33. (Northern Ireland or Northern Irish).mp.

34. (Greece or Greek).mp.

35. Iceland*.mp.

36. (Ireland or Irish).mp.

37. (Italy or Italian*).mp.

38. Luxembourg*.mp.

39. (Netherlands or Dutch).mp.

40. (Portugal or Portuguese).mp.

41. (Denmark or Danish).mp.

42. (Norway or Norwegian*).mp.

43. (Sweden or Swedish).mp.

44. (Spain or Spanish).mp.

45. (Switzerland or Swiss).mp.

46. Armenia*.mp.

47. Azerbaijan*.mp.

48. Georgia*.mp.

49. (Turkey or Turkish).mp.

50. (Malta or Maltese).mp.

51. Ulster.mp.

52. Belgian*.mp.

53. Andorra*.mp.

54. (Cyprus or Cypriot*).mp.

55. Czech*.mp.

56. (Kazakhstan or Kazakh*).mp.

57. Liechtenstein*.mp.

58. (Monaco or Monacian* or Monegasque*).mp.

59. (San Marino or Sammarinese*).mp.

60. (Vatican or Vanticanian*).mp.

America:

1. exp North America/

2. Canada.mp.

3. (US or USA).mp.

4. United States.mp.

5. America*.mp.

6. (Alabama or Alaska or Arizona or Arkansas or California or Colorado or Connecticut or Delaware or Florida or Georgia or Hawaii or Idaho or Illinois or Indiana or Iowa or Kansas or Kentucky or Louisiana or Maine or Maryland or Massachusetts or Michigan or Minnesota or Mississippi or Missouri or Montana or Nebraska or Nevada or New Hampshire or New Jersey or New Mexico or New York or North Carolina or North Dakota or Ohio or Oklahoma or Oregon or Pennsylvania or Rhode Island or South Carolina or South Dakota or Tennessee or Texas or Utah or Vermont or Virginia or Washington or West Virginia or Wisconsin or Wyoming).ti,ab.

Limits:

English language

PY(Publication Year):

Time period 1/1/2002 to 31/12/2013

PT (Publication Type):

Deselect conference papers and other publication types which will not use STROBE guidance for reporting (e. letter to the editor)

(autobiography or biography or case reports or clinical conference or clinical trial, all or clinical trial, phase i or clinical trial, phase ii or clinical trial, phase iii or clinical trial, phase iv or clinical trial or congresses or consensus development conference or consensus development conference, nih or controlled clinical trial or dictionary or directory or editorial or in vitro or interactive tutorial or interview or lectures or legal cases or legislation or letter or news or newspaper article or patient education handout or portraits or randomized controlled trial or video-audio media or webcasts)

Selection of human studies:

Animals/ not humans/

Not previous

**Table A: STROBE scoring sheet**

| **Original**  **STROBE**  **Item No.** | **STROBE Description** | **Rules/Explanatory notes** | **Answer choices** | | | | |
| --- | --- | --- | --- | --- | --- | --- | --- |
|  |  |  | **yes** | **partly** | **no** | **unclear** | **not applicable** |
| **Title and Abstract** | | | | | | | |
| 1a | Is the design described adequately in the title or abstract? | If the study design was not specifically stated, this should be recorded as not being complete. |  |  |  |  |  |
| 1b | Does the abstract provide an informative summary of what was done and found? | The abstract provides key information that enables readers to understand a study and decide whether to read the article and should only present information that is provided in the article. |  |  |  |  |  |
| **Introduction** | | | | | | | |
| 2 | Is the scientific background and rationale for the investigation reported? | Paper should give an overview of what is known on a topic and what gaps in current knowledge are addressed by the study. |  |  |  |  |  |
| 3a | Are any pre specified hypotheses reported? | Objectives are the detailed aims of the study. Well-crafted objectives specify populations, exposures and outcomes, and parameters that will be estimated. |  |  |  |  |  |
| 3b | Are the objectives reported? |  |  |  |  |  |  |
| **Methods** | | | | | | | |
| 4 | Are the key elements (ie, retrospective/prospective, cohort/cross-sectional) of the study design presented early in the paper? | For example, authors should indicate that the study was a cohort study, which followed people over a particular time period, and describe the group of persons that comprised the cohort and their exposure status. Authors should refrain from simply calling a study ‘prospective’ or ‘retrospective’ because these terms are ill defined |  |  |  |  |  |
| 5a | Are the settings reported? | Studies that did not report the setting or locations but referred readers to a previous publication should be considered as inconsistent with complete reporting. Readers need information on setting and locations to assess the context and generalizability of a study’s results. |  |  |  |  |  |
| 5b | Are the locations reported? |  |  |  |  |  |  |
| 5c | Are relevant dates including periods of recruitment reported? | Authors should state dates rather than only describing the length of time periods. If the dates of recruitment were recorded anywhere in the article (not necessarily in the “Methods” section), the corresponding item should be rated as complete. |  |  |  |  |  |
| 5d | Are relevant dates including periods of exposure reported? |  |  |  |  |  |  |
| 5e | Are relevant dates including periods of follow-up reported? |  |  |  |  |  |  |
| 5f | Are relevant dates including periods of data collection reported? |  |  |  |  |  |  |
| 6a | Are the eligibility criteria for participants described? | Detailed descriptions of the study participants help readers understand the applicability of the results. Clinical, demographic and other characteristics of eligible participants should be described. Eligibility criteria may be presented as inclusion and exclusion criteria. |  |  |  |  |  |
| 6b | Are the sources of participants described? |  |  |  |  |  |  |
| 6c | Are the methods of selection described? |  |  |  |  |  |  |
| 6d | Are the methods of follow-up described? | Knowing details about follow-up procedures, including whether procedures minimized nonresponse and loss to follow-up and whether the procedures were similar for all participants, informs judgments about the validity of results. |  |  |  |  |  |
| 6e | If it is a matched study, are the matching criteria and the numbers of exposed and unexposed described? | Because matching can be done in various ways, with one or more controls per case,the rationale for the choice of matching variables and the details of the method used should be described. To allow readers to judge whether the matched design was appropriately taken into account in the analysis, we recommend that authors describe in detail what statistical methods were used to analyse the data. |  |  |  |  |  |
| 7a | Are all outcomes described if applicable? | Disease outcomes require adequately detailed description of the diagnostic criteria. |  |  |  |  |  |
| 7b | Are all exposures described if applicable? |  |  |  |  |  |  |
| 7c | Are all predictors described if applicable? |  |  |  |  |  |  |
| 7d | Are potential confounders described? | If the confounders were recorded anywhere in the article (not necessarily in the “Methods” section), the corresponding item should be rated as complete. |  |  |  |  |  |
| 7e | Are all effect modifiers described? | Authors should declare all ‘candidate variables’ considered for statistical analysis,rather than selectively reporting only those included in the final models |  |  |  |  |  |
| 7f | Are diagnostic criteria described if applicable? |  |  |  |  |  |  |
| 8a | Are the sources of data and details of methods of measurement given for each variable of interest? | The way in which exposures, confounders and outcomes were measured affects the reliability and validity of a study. Measurement error and misclassification of exposures  or outcomes can make it more difficult to detect cause-effect relationships, or may produce spurious relationships. |  |  |  |  |  |
| 8b | If there is more than 1 group, are the measurement methods comparable? |  |  |  |  |  |  |
| 9 | Was there any effort to address potential sources of bias? | Authors should have made attempts to address sources of bias, if they incorporated any tools to do this, eg, using standardized definitions or validated scoring systems should be rated as complete. Addressing sources of bias should never be considered “not applicable” in an observational study. |  |  |  |  |  |
| 10 | Did they describe how the study size was determined? | The importance of sample size determination in observational studies depends on the context. Investigators should report pertinent formal sample size calculations if they were done. |  |  |  |  |  |
| 11a | Did they describe how quantitative variables were handled in the analysis? | Authors should explain why and how they grouped quantitative data, including the number of categories, the cut-points, and category mean or median values. Whenever data are reported in tabular form, the counts of cases, controls, persons at risk, person-time at risk, etc. should be given for each category. Tables should not consist solely of effect-measure estimates or results of model fitting. |  |  |  |  |  |
| 11b | Did they describe which groupings were chosen for quantitative variables? |  |  |  |  |  |  |
| 11c | Did they describe why quantitative groups were chosen? |  |  |  |  |  |  |
| 12a | Did they describe all statistical methods including those to deal with confounding? | In relation to statistical methods, unless authors state which confounders were adjusted for and why, should not be rated as complete. Authors should clarify reasons for particular analyses. |  |  |  |  |  |
| 12b | Did they describe methods to examine subgroups and interactions? | Readers need to know which subgroup analyses were planned in advance, and which arose while analysing the data. |  |  |  |  |  |
| 12c | Did they explain how missing data was addressed? | Authors should report the number of missing values for each variable of interest (exposures, outcomes, confounders) and for each step in the analysis. Authors should give reasons for missing values if possible, and indicate how many individuals were excluded because of missing data when describing the flow of participants through the study. |  |  |  |  |  |
| 12d | Did they explain if applicable how losses to follow-up were addressed? | Authors to report how many patients were lost to follow-up and what censoring strategies they used. |  |  |  |  |  |
| 12e | Did they describe any sensitivity analysis? | Sensitivity analyses are useful to investigate whether or not the main results are consistent with those obtained with alternative analysis strategies or assumptions |  |  |  |  |  |
| **Results** | | | | | | | |
| 13a | Did they report the numbers of individuals at each stage of the study numbers potentially eligible, examined for eligibility, confirmed eligible, included in the study, and completed follow-up and were analysed? | Those included in a study often differ in relevant ways from the target population to which results are applied. This may result in estimates of prevalence or incidence that do not reflect the experience of the target population. |  |  |  |  |  |
| 13b | Did they give reasons for nonparticipation at each stage? ^a^ | Explaining the reasons why people no longer participated in a study or why they were excluded from statistical analyses helps readers judge whether the study population was representative of the target population and whether bias was possibly introduced. |  |  |  |  |  |
| 13c | Did they use a flow diagram if appropriate? |  |  |  |  |  |  |
| 14a | Did they give the characteristics of study participants (eg, demographic, clinical, and social) and information on exposures and potential confounders? | Readers need descriptions of study participants and their exposures to judge the generalizability of the findings. Information about potential confounders, including whether and how they were measured, influences judgments about study validity. Authors should give the mean and Standard deviation, or when the data have an asymmetrical distribution, as is often the case, the median and percentile range (eg, 25th and 75th percentiles). |  |  |  |  |  |
| 14b | Did they indicate the number of participants with missing data for each variable of interest? | As missing data may bias or affect generalizability of results, authors should tell readers amounts of missing data for exposures, potential confounders, and other important characteristics of patients. Should also include the extent of loss to follow-up. |  |  |  |  |  |
| 14c | Did they summarize follow-up time (average and total amount)? | Readers need to know the duration and extent of follow-up for the available outcome data. |  |  |  |  |  |
| 15a | Did they report numbers of outcome measures over time? | Authors should report the numbers of events for each outcome of interest. Consider reporting the event rate per person-year of follow-up. If the risk of an event changes over follow-up time, present the numbers and rates of events in appropriate intervals of follow-up or as a Kaplan-Meier life table or plot. |  |  |  |  |  |
| 15b | Did they report summary measures over time? |  |  |  |  |  |  |
| 16a | Did they give unadjusted estimates and, if applicable, confounder-adjusted estimates and their precision (eg, 95% confidence interval)? | Readers can compare unadjusted measures of association with those adjusted for potential confounders and judge by how much, and in what direction, they changed. |  |  |  |  |  |
| 16b | Did they detail which confounders were adjusted for and why they were included? | Authors should explain all potential confounders considered,and the criteria for excluding or including variables in statistical models. Decisions about excluding or including variables should be guided by knowledge, or explicit assumptions, on causal relations. |  |  |  |  |  |
| 16c | Did they report category boundaries when continuous variables were categorized? | Authors should report the category boundaries; and report the range of the data and the mean or median values within categories. |  |  |  |  |  |
| 16d | Did they, if relevant, consider translating estimates of relative risk into absolute risk for a meaningful period? | it was only appropriate to translate relative risk into absolute risk if there was convincing evidence of a causal association. |  |  |  |  |  |
| 17a | Did they report on other analyses done, eg, analysis of subgroups or interactions? | Authors should report which analyses were planned, and which were not. This will allow readers to judge the implications of multiplicity, taking into account the study’s position on the continuum from discovery to verification or refutation. |  |  |  |  |  |
| 17b | Did they do a sensitivity analysis? | Sensitivity analyses are helpful to investigate the influence of choices made in the statistical analysis, or to investigate the robustness of the findings to missing data or possible biases. |  |  |  |  |  |
| **Discussion** | | | | | | | |
| 18 | Did they summarize key results with reference to study objectives? | The short summary reminds readers of the main findings and may help them assess whether the subsequent interpretation and implications offered by the authors are supported by the findings. |  |  |  |  |  |
| 19 | Did they discuss the limitations of the study taking into account potential sources of bias or imprecision (including discussion of the magnitude of any potential sources of bias)? | Authors should identify the sources of bias and confounding that could have affected results, but also to discuss the relative importance of different biases, including the likely direction and magnitude of any potential bias. Authors may compare the study being presented with other studies in the literature in terms of validity, generalizability and precision. In this approach, each study can be viewed as contribution to the literature, not as a stand-alone basis for inference and action. |  |  |  |  |  |
| 20 | Did they give a cautious overall interpretation of results considering objectives, limitations, multiplicity of analyses, results from similar studies, and other relevant evidence? | Authors should consider potential sources of bias, including loss to follow-up and non-participation. Due consideration should be given to confounding, the results of relevant sensitivity analyses, and to the issue of multiplicity and subgroup analyses. Authors should also consider residual confounding due to unmeasured variables or imprecise measurement of confounders. Authors should put their results in context with similar studies and explain how the new study affects the existing body of evidence, by referring to a systematic review. |  |  |  |  |  |
| 21 | Did they discuss the generalizability (external validity) of the study results? | Can results be applied to an individual, groups or populations that differ from those enrolled in the study with regard to age, sex, ethnicity, severity of disease, and co-morbid conditions? Are the nature and level of exposures comparable, and the definitions of outcomes relevant to another setting or population? Are results from health services research in one country applicable to health systems in other countries? |  |  |  |  |  |
| **Other Information** | | | | | | | |
| 22a | Did they give the source of the funding in the present study and, if applicable, for the original study on which the present article is based? |  |  |  |  |  |  |
| 22b | Did they give the role of the funders in the present study and, if applicable, for the original study on which the present article is based? | Unless the role of the funders was specifically stated this should not be recorded as complete. |  |  |  |  |  |

^a^ For studies based on disease registries or databases, a number of the checklist items are not applicable, eg, the dates of recruitment, numbers eligible at each stage of the study, reasons for nonparticipation, or flow diagrams

The number and proportion of reported items (“yes” & “partly” responses) and not reported items (all responses except “yes”, “partly” or “not applicable”) will be analysed for each study

**Item B: List of articles included for review by date of publication**

1. Fried LF, Shlipak MG, Crump C, et al. Renal insufficiency as a predictor of cardiovascular outcomes and mortality in elderly individuals. Journal of the American College of Cardiology. 2003;41:1364-1372.

2. Drey N, Roderick P, Mullee M, Rogerson M. A population-based study of the incidence and outcomes of diagnosed chronic kidney disease. American Journal of Kidney Diseases. 2003;42:677-684.

3. Foley RN, Murray AM, Li S, et al. Chronic Kidney Disease and the Risk for Cardiovascular Disease, Renal Replacement, and Death in the United States Medicare Population, 1998 to 1999. Journal of the American Society of Nephrology. 2005;16:489-495.

4. Shlipak MG, Fried LF, Cushman M, et al. Cardiovascular mortality risk in chronic kidney disease: Comparison of traditional and novel risk factors. JAMA. 2005;293:1737-1745.

5. Fried LF, Katz R, Sarnak MJ, et al. Kidney Function as a Predictor of Noncardiovascular Mortality. Journal of the American Society of Nephrology. 2005;16:3728-3735.

6. O’Hare AM, Bertenthal D, Covinsky KE, et al. Mortality Risk Stratification in Chronic Kidney Disease: One Size for All Ages? Journal of the American Society of Nephrology. 2006;17:846-853.

7. Hallan SI, Dahl K, Oien CM, et al. Screening strategies for chronic kidney disease in the general population: follow-up of cross sectional health survey. BMJ. 2006;333:1047.

8. Wong CF, McCarthy M, Howse MLP, Williams PS. Factors Affecting Survival in Advanced Chronic Kidney Disease Patients Who Choose Not to Receive Dialysis. Renal Failure. 2007;29:653-659.

9. O'Hare AM, Choi AI, Bertenthal D, et al. Age Affects Outcomes in Chronic Kidney Disease. Journal of the American Society of Nephrology. 2007;18:2758-2765.

10. De Biase V, Tobaldini O, Boaretti C, et al. Prolonged conservative treatment for frail elderly patients with end-stage renal disease: the Verona experience. Nephrology Dialysis Transplantation. 2008;23:1313-1317.

11. Hallan S, Astor B, Romundstad S, Aasarød K, Kvenild K, Coresh J. Association of kidney function and albuminuria with cardiovascular mortality in older vs younger individuals: The hunt ii study. Archives of Internal Medicine. 2007;167:2490-2496.

12. Pizzarelli F, Lauretani F, Bandinelli S, et al. Predictivity of survival according to different equations for estimating renal function in community-dwelling elderly subjects. Nephrology Dialysis Transplantation. 2009;24:1197-1205.

13. Nitsch D, Mylne A, Roderick PJ, Smeeth L, Hubbard R, Fletcher A. Chronic kidney disease and hip fracture-related mortality in older people in the UK. Nephrology Dialysis Transplantation. 2009;24:1539-1544.

14. Snyder JJ, Collins AJ. Association of Preventive Health Care with Atherosclerotic Heart Disease and Mortality in CKD. Journal of the American Society of Nephrology. 2009;20:1614-1622.

15. Roderick PJ, Atkins RJ, Smeeth L, et al. CKD and Mortality Risk in Older People: A Community-Based Population Study in the United Kingdom. American Journal of Kidney Diseases. 2009;53:950-960.

16. Seyfarth M, Kastrati A, Mann JFE, et al. Prognostic Value of Kidney Function in Patients With ST-Elevation and Non–ST-Elevation Acute Myocardial Infarction Treated With Percutaneous Coronary Intervention. American Journal of Kidney Diseases. 2009;54:830-839.

17. Carson RC, Juszczak M, Davenport A, Burns A. Is Maximum Conservative Management an Equivalent Treatment Option to Dialysis for Elderly Patients with Significant Comorbid Disease? Clinical Journal of the American Society of Nephrology. 2009;4:1611-1619.

18. El-Ghoul B, Elie C, Sqalli T, et al. Nonprogressive Kidney Dysfunction and Outcomes in Older Adults with Chronic Kidney Disease. Journal of the American Geriatrics Society. 2009;57:2217-2223.

19. Demoulin N, Beguin C, Labriola L, Jadoul M. Preparing renal replacement therapy in stage 4 CKD patients referred to nephrologists: a difficult balance between futility and insufficiency. A cohort study of 386 patients followed in Brussels. Nephrology Dialysis Transplantation. 2011;26:220-226.

20. Pilotto A SD, Franceschi M, Aucella F, D'Ambrosio P, Scarcelli C, Ferrucci L. A multidimensional approach to the geriatric patient with chronic kidney disease. Journal of Nephrology. 2010;23:5-10.

21. Dalrymple L, Katz R, Kestenbaum B, et al. Chronic Kidney Disease and the Risk of End-Stage Renal Disease versus Death. J GEN INTERN MED. 2011;26:379-385.

22. Andrea Corsonello CP, Fabrizia Lattanzio, Sabrina Garasto, Francesco Corica, Silvia Bustacchini, Enrico E. Guffanti, Angela M. Abbatecola, Vincenzo Mari, Filippo L. Fimognari, and Raffaele Antonelli Incalzi. . Does Concealed Chronic Kidney Disease Predict Survival of Older Patients Discharged from Acute Care Hospitals? Rejuvenation Research. 2010;13:539-545.

23. Muntner P, Bowling CB, Gao L, et al. Age-Specific Association of Reduced Estimated Glomerular Filtration Rate and Albuminuria with All-Cause Mortality. Clinical Journal of the American Society of Nephrology. 2011;6:2200-2207.

24. Heras M F-RM, Sánchez R, Guerrero MT, Molina A, Rodríguez MA, Alvarez-Ude F. Elderly patients with chronic kidney disease: What happens after five years of follow-up? Nefrologia. 2012;32:300-305.

25. Drion I, van Hateren KJJ, Joosten H, et al. Chronic kidney disease and mortality risk among older patients with type 2 diabetes mellitus (ZODIAC-24). Age and Ageing. 2012;41:345-350.

26. Marks A, Black C, Fluck N, et al. Translating chronic kidney disease epidemiology into patient care—the individual/public health risk paradox. Nephrology Dialysis Transplantation. 2012;27:iii65-iii72.

27. Ahmed A, Fonarow GC, Zhang Y, et al. Renin-Angiotensin Inhibition in Systolic Heart Failure and Chronic Kidney Disease. The American Journal of Medicine. 2012;125:399-410.

28. Binder EF, White HK, Resnick B, McClellan WM, Lei L, Ouslander JG. A Prospective Study of Outcomes of Nursing Home Residents with Chronic Kidney Disease with and without Anemia. Journal of the American Geriatrics Society. 2012;60:877-883.

29. Navaneethan SD, Schold JD, Arrigain S, et al. Serum triglycerides and risk for death in Stage 3 and Stage 4 chronic kidney disease. Nephrology Dialysis Transplantation. 2012;27:3228-3234.

30. Hemmelgarn BR, James MT, Manns BJ, et al. RAtes of treated and untreated kidney failure in older vs younger adults. JAMA. 2012;307:2507-2515.

31. Dobre M, Brateanu A, Rashidi A, Rahman M. Electrocardiogram Abnormalities and Cardiovascular Mortality in Elderly Patients with CKD. Clinical Journal of the American Society of Nephrology. 2012;7:949-956.

32. Shastri S, Katz R, Rifkin DE, et al. Kidney Function and Mortality in Octogenarians: Cardiovascular Health Study All Stars. Journal of the American Geriatrics Society. 2012;60:1201-1207.

33. Marks A, MacLeod C, McAteer A, et al. Chronic kidney disease, a useful trigger for proactive primary care? Mortality results from a large UK cohort. Family Practice. 2013;30:282-289.

34. Ahmed A, Rich MW, Zile M, et al. Renin-Angiotensin Inhibition in Diastolic Heart Failure and Chronic Kidney Disease. The American Journal of Medicine. 2013;126:150-161.

35. Westerberg P-A, Tivesten Å, Karlsson MK, et al. Fibroblast growth factor 23, mineral metabolism and mortality among elderly men (Swedish MrOs). BMC nephrology. 2013;14:85-85.

36. Faller B, Beuscart J-B, Frimat L. Competing-risk analysis of death and dialysis initiation among elderly (≥80 years) newly referred to nephrologists: a French prospective study. BMC Nephrology. 2013;14:103-103.

37. Huang X, Jiménez-Moleón JJ, Lindholm B, et al. Mediterranean Diet, Kidney Function, and Mortality in Men with CKD. Clinical Journal of the American Society of Nephrology. 2013;8:1548-1555.

**Table B:** Summary of simple and weighted Kappa coefficient, measuring agreement between reviewers for the NOS, SIGN and CASP tool.

|  | Kappa | Agreement | P value |
| --- | --- | --- | --- |
| NOS (weighted) | | | |
| Reviewer 1 vs Reviewer 2 | 0.18 | Poor | 0.12 |
| Reviewer 1 vs Reviewer 3 | 0.28 | Fair | 0.004 |
| Reviewer 1 vs Reviewer 4 | 0.12 | Poor | 0.02 |
| SIGN (unweighted) | | | |
| Does not apply vs Everything else grouped | | | |
| Reviewer 1 vs Reviewer 2 | -0.02 | poor | 0.87 |
| Reviewer 1 vs Reviewer 3 | 0.05 | poor | 0.51 |
| Reviewer 1 vs Reviewer 4 | 0.19 | poor | <0.001 |
|  | | | |
| Can't say vs No/Yes grouped | | | |
| Reviewer 1 vs Reviewer 2 | -0.04 | poor | 0.62 |
| Reviewer 1 vs Reviewer 3 | 0.47 | moderate | <0.001 |
| Reviewer 1 vs Reviewer 4 | -0.04 | poor | 0.49 |
|  | | | |
| No vs Yes | | | |
| Reviewer 1 vs Reviewer 2 | 0.31 | Fair | 0.004 |
| Reviewer 1 vs Reviewer 3 | 0.51 | Moderate | <0.001 |
| Reviewer 1 vs Reviewer 4 | 0.24 | Fair | <0.001 |
| CASP (unweighted) | | | |
| Can't tell vs No/Yes grouped | | | |
| Reviewer 1 vs Reviewer 2 | 0.90 | Very good | 0.30 |
| Reviewer 1 vs Reviewer 3 | 0.72 | good | <0.001 |
| Reviewer 1 vs Reviewer 4 | 0.05 | poor | 0.05 |
|  | | | |
| Yes vs No | | | |
| Reviewer 1 vs Reviewer 2 | 0.69 | good | <0.001 |
| Reviewer 1 vs Reviewer 3 | -0.06 | poor | 0.64 |
| Reviewer 1 vs Reviewer 4 | -0.07 | poor | 0.40 |
